# Laboratory evaluation of the miniature direct-on-blood PCR nucleic acid lateral flow immunoassay (mini-dbPCR-NALFIA), a simplified molecular diagnostic test for *Plasmodium*

**DOI:** 10.1186/s12936-023-04496-4

**Published:** 2023-03-17

**Authors:** Norbert J. van Dijk, Sandra Menting, Ellen M. S. Wentink-Bonnema, Patricia E. Broekhuizen-van Haaften, Elen Withycombe, Henk D. F. H. Schallig, Petra F. Mens

**Affiliations:** 1 Department of Medical Microbiology and Infection Prevention, Experimental Parasitology. Meibergdreef 9, Amsterdam University Medical Centres, Academic Medical Centre at the University of Amsterdam, 1105 AZ Amsterdam, The Netherlands; 2 Amsterdam Institute for Infection and Immunity, Infectious Diseases Programme, Amsterdam, The Netherlands; 3 Department of Medical Microbiology and Infection Prevention, Clinical Parasitology. Meibergdreef 9, Amsterdam University Medical Centres, Academic Medical Centre at the University of Amsterdam, 1105 AZ Amsterdam, The Netherlands; 4 Abingdon Health. York Biotech Campus, Sand Hutton, York, YO41 1LZ UK

**Keywords:** Malaria, *Plasmodium*, Simplified molecular diagnostics, PCR, miniPCR, Nucleic Acid Lateral Flow Immunoassay, Laboratory evaluation

## Abstract

**Background:**

Point-of-care diagnosis of malaria is currently based on microscopy and rapid diagnostic tests. However, both techniques have their constraints, including poor sensitivity for low parasitaemias. Hence, more accurate diagnostic tests for field use and routine clinical settings are warranted. The miniature direct-on-blood PCR nucleic acid lateral flow immunoassay (mini-dbPCR-NALFIA) is an innovative, easy-to-use molecular assay for diagnosis of malaria in resource-limited settings. Unlike traditional molecular methods, mini-dbPCR-NALFIA does not require DNA extraction and makes use of a handheld, portable thermal cycler that can run on a solar-charged power pack. Result read-out is done using a rapid lateral flow strip enabling differentiation of *Plasmodium falciparum* and non-*falciparum* malaria infections. A laboratory evaluation was performed to assess the performance of the mini-dbPCR-NALFIA for diagnosis of pan-*Plasmodium* and *P. falciparum* infections in whole blood.

**Methods:**

Diagnostic accuracy of the mini-dbPCR-NALFIA was determined by testing a set of *Plasmodium*-positive blood samples from returned travellers (n = 29), and *Plasmodium*-negative blood samples from travellers with suspected malaria (n = 23), the Dutch Blood Bank (n = 19) and intensive care patients at the Amsterdam University Medical Centers (n = 16). Alethia Malaria (LAMP) with microscopy for species differentiation were used as reference. Limit of detection for *P. falciparum* was determined by 23 measurements of a dilution series of a *P. falciparum* culture. A fixed sample set was tested three times by the same operator to evaluate the repeatability, and once by five different operators to assess the reproducibility.

**Results:**

Overall sensitivity and specificity of the mini-dbPCR-NALFIA were 96.6% (95% CI, 82.2%–99.9%) and 98.3% (95% CI, 90.8%–100%). Limit of detection for *P. falciparum* was 10 parasites per microlitre of blood. The repeatability of the assay was 93.7% (95% CI, 89.5%–97.8%) and reproducibility was 84.6% (95% CI, 79.5%–89.6%).

**Conclusions:**

Mini-dbPCR-NALFIA is a sensitive, specific and robust method for molecular diagnosis of *Plasmodium* infections in whole blood and differentiation of *P. falciparum*. Incorporation of a miniature thermal cycler makes the assay well-adapted to resource-limited settings. A phase-3 field trial is currently being conducted to evaluate the potential implementation of this tool in different malaria transmission areas.

## Background

Correct and timely diagnosis of malaria is key in the management and control of this disease. Traditionally, microscopy of Giemsa-stained thick and thin blood film has been the standard diagnostic technique applied in endemic settings. Although it is able to differentiate the causative *Plasmodium* species, its sensitivity for low parasite densities is limited and adequate slide reading requires extensive training and experience [1–4]. The development of rapid diagnostic tests (RDTs) has brought a fast and easy-to-use alternative for malaria diagnosis. Since their introduction, RDTs have proven to be an essential tool for malaria control in remote endemic regions [5]. However, they usually do not detect < 100 parasites per microliter of blood, which makes them of limited use in near-elimination areas where such low parasite counts are often prevalent [6–8]. False-negative RDT results can also arise for *P. falciparum* strains with a genetic deletion for the antigen targeted by RDTs, histidine-rich protein 2 (HRP2). Over the past decade, this genotype has become widespread in South America, and increasing prevalence has now been reported for African and Asian countries as well [9–12]. Conversely, residual parasite antigen in the blood after treatment and complete parasite clearance is frequently observed and may result in false-positive RDT diagnosis [13, 14].

The limitations of microscopy and RDTs can be overcome by the use of nucleic acid amplification techniques (NAATs) [15]. Examples are endpoint polymerase chain reaction (PCR) and real-time quantitative PCR (qPCR), techniques that are commonly applied for malaria diagnosis and research in high-resource settings [16–19]. However, the requirement of well-trained laboratory personnel as well as expensive PCR machines that rely on a stable power source, restrict the use of NAATs in malaria-endemic countries. An alternative to PCR is loop-mediated isothermal amplification (LAMP), a simplified molecular assay with an easy readout that makes use of isothermal DNA amplification [20]. Nevertheless, current LAMP formats are generally unsuited for multiplex amplification, hampering *Plasmodium* species differentiation [21].

**Fig. 1:**
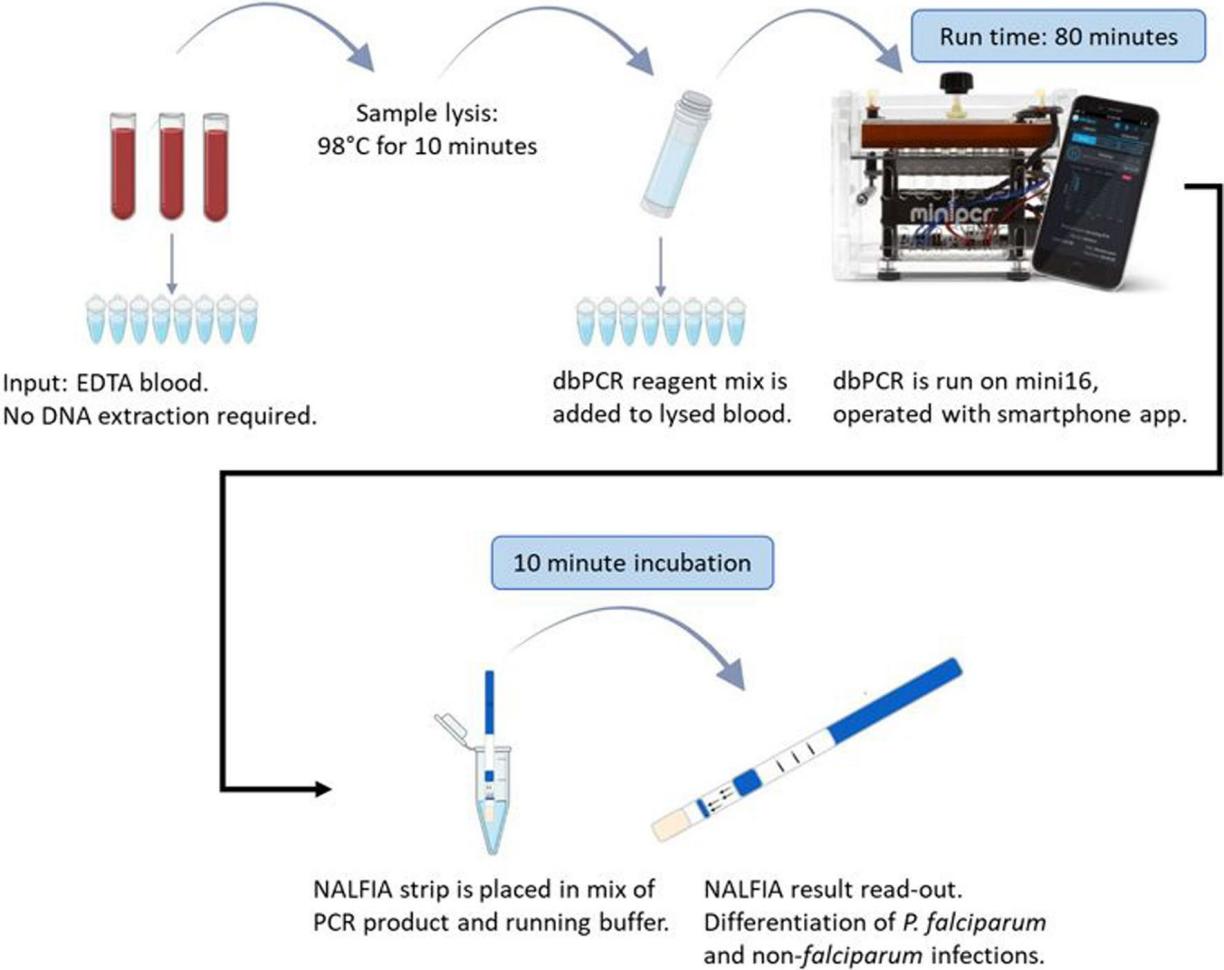
Workflow of the mini-dbPCR-NALFIA

Consequently, there is still a need for a highly sensitive, user-friendly and field-deployable diagnostic test for malaria that can discriminate *Plasmodium* species. An innovative assay has recently been developed to meet these requirements: the miniature direct-on-blood PCR nucleic acid lateral flow immunoassay (mini-dbPCR-NALFIA, Fig. 1). This platform combines three techniques to overcome the issues encountered when attempting to implement traditional PCR methods in limited-resource settings. First of all, the direct-on-blood PCR (dbPCR) uses a specialized reagent mix that eliminates the need of DNA extraction prior to amplification [22, 23]. Instead, the PCR can be performed directly on a template of EDTA-anticoagulated whole blood. The dbPCR also has a duplex format which can detect all (pan) *Plasmodium* species infecting humans and differentiate *P. falciparum* infections. The second innovative element is the use of a miniature thermal cycler to run the dbPCR, called miniPCR (miniPCR bio, Massachusetts, USA). It is a hand-held, portable device that can be programmed with a smartphone or laptop application, either through USB cable or Bluetooth connection. The latest model, mini16, has an affordable price of approximately 800 USD (compared to 3000–5000 USD for a conventional PCR thermal cycler) and can process 16 samples per run. The mini16 can run on mains power, but also on a portable and solar-chargeable power pack, making the system completely autonomous and suitable for rural or emergency settings with unstable or no electricity supply. Finally, the result of the dbPCR is easily and rapidly read out with NALFIA, an immunochromatographic flow strip that can detect labelled PCR amplicons [22–25]. A NALFIA strip is placed in a mixture of dbPCR product and running buffer, after which the dbPCR amplicons will flow over the strip. Neutravidin-labelled carbon particles on the NALFIA strip will bind to the labelled dbPCR amplicons, and this complex is visualized within 10 min when it is captured by the two amplicon-specific antibody lines on the NALFIA strip.

Earlier prototypes of the dbPCR-NALFIA assay have shown promising results in field evaluations, with sensitivity and specificity results up to 97.2% and 95.5%, respectively, using light microscopy as reference standard, and a detection limit for *P. falciparum* infections of 1 parasite per microlitre (p/μL) of blood [22, 23]. In these studies, the dbPCR was still run on a conventional thermal cycler. By optimizing the dbPCR protocol, the mini-dbPCR-NALFIA can now be run on a miniPCR device, making the method better adapted to field settings with limited resources. This article describes the laboratory evaluation of the optimized mini-dbPCR-NALFIA as a multiplex assay for the detection of pan-*Plasmodium* and *P. falciparum* infections in blood.

## Methods

### Direct-on-blood PCR reagent mix

The dbPCR is a duplex reaction targeting two regions in the *Plasmodium* 18S rRNA gene: one that is highly conserved in the genus *Plasmodium* (the pan-*Plasmodium* target), and a second that is specific for *P. falciparum* [26, 27]. By using 5’-labelled primer pairs (Eurogentec, Liège, Belgium) previously described in literature, both target amplicons will carry a biotin label and a target-specific label (Table 1) [23]. The dbPCR reagent mix consists of 10 μL of 2× Phusion Blood PCR buffer (Thermo Fisher Scientific, Waltham, MA, USA), 0.1 μL of Phire Hot Start II DNA polymerase (Thermo Fisher Scientific), labelled primers and sterile water to make a total volume of 22.5 μL per sample.

**Table 1:** Primer sequences, labels and final concentrations used in the direct-on-blood PCR

Primer	Sequence	5′ labelling	Final concentration (nM)
Pan-*Plasmodium* Forward	5′-TCAGATACCGTCGTAATCTTA-3′	Dig	250
Pan-*Plasmodium* Reverse	5′-AACTTTCTCGCTTGCGCG-3′	Biotin	250
*P. falciparum* Forward	5′-GTAATTGGAATGATAGGAATTTACAAGGT-3′	FAM	75
*P. falciparum* Reverse	5′-TCAACTACGAACGTTTTAACTGCAAC-3′	Biotin	62.5

*Dig* digoxigenin, *FAM* fluorescein amidite

### Direct-on-blood PCR on miniature thermal cycler

The template format for the dbPCR is 2.5 μL of EDTA-anticoagulated blood. Every mini-dbPCR-NALFIA run includes controls, which are a *P. falciparum*-infected EDTA blood sample and a *Plasmodium*-negative EDTA blood sample. As a first step, the samples were lysed at 98 °C for 10 min on the mini16 thermal cycler (miniPCR bio, Massachusetts, USA), a miniature endpoint PCR device (dimensions: 5 × 13 × 10 cm, weight: 0.5 kg) which can also be used for heat block protocols. The miniPCR smartphone application was used to programme the lysis protocol on the mini16 device through Bluetooth connection. After the lysis of the EDTA blood templates, 22.5 μL of the dbPCR reagent mix was added to each (total reaction volume 25 μL). The dbPCR was also run on the mini16 thermal cycler. Its protocol consisted of an initial activation step of 1 min at 98 °C, followed by 10 cycles of 5 s at 98 °C, 15 s at 61 °C and 30 s at 72 °C; next, 28 cycles of 5 s at 98 °C, 15 s at 58 °C and 30 s at 72 °C; and a final extension step of 72 °C for 2 min.

### Read-out with NALFIA

**Fig. 2:**
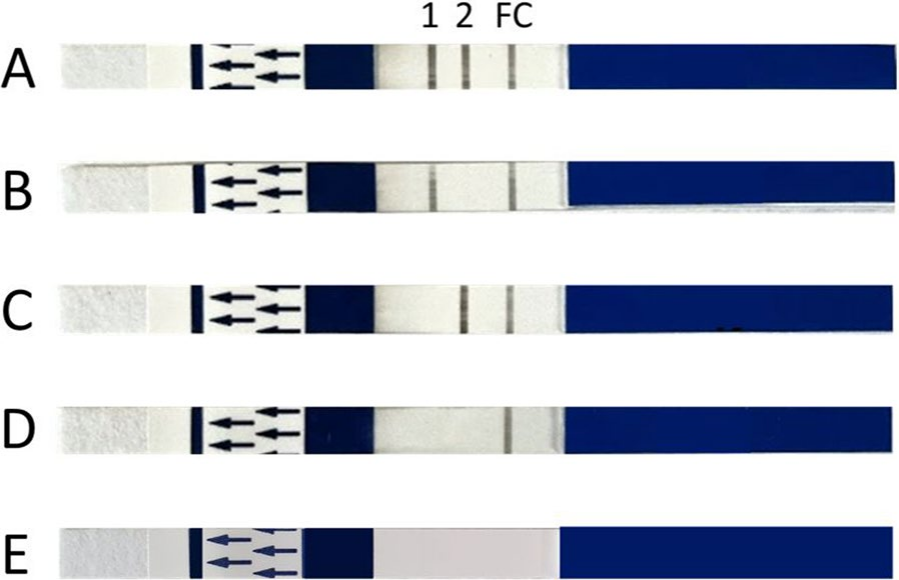
Possible NALFIA results after dbPCR for pan-*Plasmodium* and *P. falciparum*. The strip has 3 potential lines that can appear: a pan-*Plasmodium* test line (1), a *P. falciparum* test line (2) and a flow control line (FC). **A** Positive pan-*Plasmodium* and *P. falciparum* test lines, thus a *P. falciparum* infection or a mixed infection including *P. falciparum*; **B** Pan-*Plasmodium* positive test line, but negative for the *P. falciparum* test line. This is a *Plasmodium* infection with a non-*falciparum* species (e.g. *P*. *vivax*); **C** Pan-*Plasmodium* negative, but a positive *P. falciparum* line. This result is also considered positive for *P. falciparum*; **D** The NALFIA has a flow control line but the pan-*Plasmodium* and *P. falciparum* test lines are absent. This is a *Plasmodium*-negative test result; **E** Pan-*Plasmodium* and *P. falciparum* negative, but also negative for the flow control line. This NALFIA test is invalid

Read-out of the results was done with NALFIA (Abingdon Health, York, UK). The test strip consists of a sample absorption pad, a conjugate pad with neutravidin-labelled carbon binding to the amplicons’ biotin label, and a nitrocellulose membrane coated with anti-digoxigenin (Dig) and anti-fluorescein isothiocyanate (FITC) antibody lines detecting and visualizing the amplicon-carbon complex. A third line on the membrane functions as a flow control (Fig. 2). After completion of the dbPCR run on the mini16, a NALFIA strip was placed in a tube with 10 μL of dbPCR product and 140 μL running buffer. After a 10 min incubation, the NALFIA results were read out. When the first line directed against the Dig-labelled pan-*Plasmodium* amplicon was positive, it indicated the presence of *Plasmodium* infection. If the second anti-FITC test line for the fluorescein amidite (FAM)-labelled *P. falciparum* amplicon was also positive, the sample was infected specifically with *P. falciparum* (or a mixed infection including *P. falciparum*). A sample with a positive pan-*Plasmodium* line and an absent *P. falciparum* line was classified positive for a non-*falciparum* malaria species, i.e. *Plasmodium vivax, Plasmodium malariae, Plasmodium ovale* or *Plasmodium knowlesi*. When only the *P. falciparum* line was visible, this result* w*as interpreted to be positive for this species. A NALFIA test was considered invalid when the flow control line was absent.

### Laboratory evaluation

#### Limit of detection

The limit of detection (LoD) for the pan-*Plasmodium* and *P. falciparum* targets was determined by testing 23 aliquots of a tenfold dilution series of a FCR3 ring-stage *P. falciparum* culture. The parasite density of the culture was determined by light microscopy. Dilutions were made in *Plasmodium*-negative EDTA blood from the Dutch blood bank. Tested parasite densities ranged from 1000 to 0.1 p/μL. LoD was defined as the lowest parasite density that was detected with 90% confidence (≥ 21 of 23 runs).

#### Sensitivity and specificity

To determine the laboratory sensitivity and specificity of the mini-dbPCR-NALFIA, a set of 87 blood specimen was tested, including samples from returned Dutch travellers with suspected malaria infection, Dutch blood donors, and intensive care unit patients from the Academic Medical Centre (Amsterdam, the Netherlands). All samples were derived from a pre-established Biobank at the Laboratory for Experimental Parasitology at the Academic Medical Centre. Both blood donors and intensive care unit patients did not travel to malaria-endemic areas in the 6 months before blood collection. The malaria status of all samples had been determined previously using the Alethia Malaria assay (Meridian Bioscience, Cincinnati, USA), a highly sensitive LAMP-based method for diagnosing malaria in non-endemic settings with a detection limit of 2 p/µL for *P. falciparum* and 0.1 p/µL for *P. vivax* [28, 29]. For samples with a positive Alethia result (n = 29, all returned travellers), the infecting *Plasmodium* species had been determined with expert microscopy. This set included 23 *P. falciparum*, 3 *P. vivax*, 2 *P. ovale* and 1 *P. malariae* infections. The *P. falciparum* samples had been quantified microscopically and ranged from 10^6^ to 10^2^ p/μL; the parasite counts of the non-*falciparum* malaria samples had not been determined at the time of microscopic examination. The 58 *Plasmodium*-negative samples comprised 19 samples from the Dutch blood donors, 16 samples from intensive care unit patients and 23 samples from malaria-suspected returned travellers with a negative Alethia diagnosis. The operator that tested all samples with mini-dbPCR-NALFIA was blinded to the reference test outcomes.

#### Accordance and concordance

Accordance and concordance are measures to express, respectively, the repeatability (intra-operator variability) and reproducibility (inter-operator variability) of qualitative tests [30, 31]. To evaluate the accordance and concordance of the mini-dbPCR-NALFIA, a single individual prepared 8 aliquots of a dilution series of FCR3 ring-stage *P. falciparum* culture and five *Plasmodium*-negative blood samples. For the accordance assessment, one operator tested three sets of aliquots with mini-dbPCR-NALFIA on three consecutive days, using the same equipment and dbPCR reagent batch numbers. To determine the concordance of the mini-dbPCR-NALFIA, five different operators from the same laboratory each tested a set of sample aliquots once. All five operators were blinded to the nature of the samples and used the same equipment and dbPCR reagent batch numbers.

### Statistical analysis

Sensitivity and specificity were calculated for the pan-*Plasmodium* target, the *P. falciparum* target and the overall assay. The Clopper-Pearson Exact method was used to calculate the 95% confidence interval (CI) of the sensitivity and specificity. Accordance and concordance were calculated in random framework, using the formulae proposed by Van der Voet and Van Raamsdonk (2004): ${ACC}_{random}=\frac{1}{L}{\sum }_{i}({{p}_{0,i}}^{2}+{{p}_{1,i}}^{2}+{{p}_{2,i}}^{2}{+{p}_{3,i}}^{2})$, where L represents the number of tested samples, p_0_ the proportion of negative results, p_1_ the proportion of pan-*Plasmodium* single positive results (i.e. only the pan line), p_2_ the proportion of *P. falciparum* single positive results (i.e. only the *P. falciparum* line) and p_3_ the proportion of double positive results (i.e. both pan and *P. falciparum* lines), for a particular sample i. For the random concordance, the following formula was used: ${CON}_{random}={{P}_{0,i}}^{2}+{{P}_{1,i}}^{2}+{{P}_{2,i}}^{2}+{{P}_{3,i}}^{2}$, where ${P}_{0,i}=\frac{1}{L}{\sum }_{i}^{L}{p}_{0,i}$,${P}_{1,i}=\frac{1}{L}{\sum }_{i}^{L}{p}_{1,i}$, ${P}_{2,i}=\frac{1}{L}{\sum }_{i}^{L}{p}_{2,i}$ and${P}_{3,i}=\frac{1}{L}{\sum }_{i}^{L}{p}_{3,i}$. Here, L represents the number of different operators, and p_0,i_, p_1,i_, p_2,i_ and p_3,i_ represent the proportion of negative, pan single positive, *P. falciparum* single positive and double positive results for a particular operator i [31]. The 95% CI of the accordance and concordance estimates was calculated by means of bootstrapping [30, 32].

## Results

### Limit of detection

The results of the *P. falciparum* culture dilution series testing are displayed in Table 2. At a confidence level of 90%, LoD was determined to be 100 p/µL for the pan-*Plasmodium* test line and 10 p/µL for the *P. falciparum* line.

**Table 2:** Results of the mini-dbPCR-NALFIA for repeated measurements of a FCR3 *P. falciparum* culture dilution in blood, in order to determine the limit of detection

Samples	No. of repetitions	Frequency of mini-dbPCR-NALFIA result per parasite density			
		Negative	Only positive for pan-*Plasmodium* line	Only positive for *P. falciparum* line	Positive for pan-*Plasmodium* and *P. falciparum* lines
*P. falciparum parasites in blood*					
1000 p/µL	23	0	0	0	23 (100%)
100 p/µL	23	0	0	0	23 (100%)
10 p/µL	23	2 (9%)	0	7 (30%)	14 (61%)
5 p/µL	23	15 (65%)	0	7 (30%)	1 (4%)
2 p/µL	23	18 (78%)	0	5 (22%)	0
1 p/µL	23	19 (83%)	0	4 (17%)	0
0.1 p/µL	23	23 (100%)	0	0	0

### Sensitivity and specificity

Of the 29 *Plasmodium* samples, 28 tested positive for the pan-*Plasmodium* line in the mini-dbPCR-NALFIA, while 1 *P. vivax* sample was false-negative for this line. All 23 *P. falciparum* samples also showed the *P. falciparum* test line. 57 *Plasmodium*-negative blood samples were negative for both test lines with mini-dbPCR-NALFIA; 1 sample from a Dutch blood donor was false-positive for the *P. falciparum* line. None of the test samples had an invalid NALFIA result.

This resulted in a sensitivity of 96.6% (95% CI, 82.2%–99.9%) and a specificity of 100% (95% CI, 93.8%–100%) for the pan-*Plasmodium* line. The sensitivity of the *P. falciparum* test line was calculated to be 100% (95% CI, 85.2%–100%), and its specificity 98.4% (95% CI, 91.6%–100%)*.* When the results of the two NALFIA test lines were combined, there were three possible outcomes: a non-*falciparum* infection, a *P. falciparum* infection and *Plasmodium*-negative. This approach resulted in an overall sensitivity of 96.6% (95% CI, 82.2%–99.9%) and specificity of 98.3% (95% CI, 90.8%–100%) of the mini-dbPCR-NALFIA.

### Accordance and concordance

An overview of the accordance test results for the mini-dbPCR-NALFIA is shown in Table 3*.* The overall accordance of all tested samples in a random framework was 93.7% (95% CI, 89.5%–97.8%).

**Table 3:** Results of the mini-dbPCR-NALFIA for a sample set tested three times by the same operator

Samples	No. of repetitions	Frequency of mini-dbPCR-NALFIA result			
		Negative	Only positive for pan-*Plasmodium* line	Only positive for *P. falciparum* line	Positive for pan-*Plasmodium* and *P. falciparum* lines
*P. falciparum parasites in blood*					
100,000 p/µL	3	0	0	0	3
10,000 p/µL	3	0	0	0	3
1000 p/µL	3	0	0	0	3
100 p/µL	3	0	0	0	3
10 p/µL	3	0	0	1	2
5 p/µL	3	1	0	2	0
2 p/µL	3	0	0	3	0
1 p/µL	3	0	0	3	0
0.1 p/µL	3	3	0	0	0
*Blood from Dutch healthy donors*					
Blood donor 1	3	3	0	0	0
Blood donor 2	3	3	0	0	0
Blood donor 3	3	3	0	0	0
Blood donor 4	3	3	0	0	0
Blood donor 5	3	3	0	0	0

Table 4 summarizes the test results for the five different operators of the mini-dbPCR-NALFIA. Based on these data, the random concordance was calculated to be 84.6% (95% CI, 79.5%–89.6%).

**Table 4:** Results of the mini-dbPCR-NALFIA for a sample set tested by five different operators

Samples	No. of repetitions	Frequency of mini-dbPCR-NALFIA result			
		Negative	Only positive for pan-*Plasmodium* line	Only positive for *P. falciparum* line	Positive for pan-*Plasmodium* and *P. falciparum* lines
*P. falciparum parasites in blood*					
100,000 p/µL	5	0	0	0	5
10,000 p/µL	5	0	0	0	5
1000 p/µL	5	0	0	0	5
100 p/µL	5	0	0	0	5
10 p/µL	5	1	0	1	3
5 p/µL	5	3	0	1	1
2 p/µL	5	3	0	1	1
1 p/µL	5	2	0	3	0
0.1 p/µL	5	5	0	0	0
*Blood from Dutch healthy donors*					
Blood donor 1	5	5	0	0	0
Blood donor 2	5	5	0	0	0
Blood donor 3	5	5	0	0	0
Blood donor 4	5	5	0	0	0
Blood donor 5	5	5	0	0	0

## Discussion

This study demonstrates that the mini-dbPCR-NALFIA is a robust, highly sensitive and specific tool for molecular diagnosis of malaria. It has a simpler workflow than traditional NAATs and requires much less resources. By incorporating the mini16 as portable, battery-powered thermal cycler, the mini-dbPCR-NALFIA can be used even in remote healthcare settings without an extensive laboratory infrastructure or stable power supply.

With an excellent overall sensitivity of 96.6% and specificity of 98.3%, the diagnostic accuracy of the mini-dbPCR-NALFIA is similar to that of traditional molecular techniques for malaria diagnosis, such as conventional PCR, qPCR and nested PCR [15]. One *P. vivax* sample gave a false-negative result. This may have been due to a low parasite density, which is common in *P. vivax* infections [33]. Unfortunately, whether this was indeed the case for this sample was unknown, as its parasitaemia had not been determined with microscopy at the time of diagnosis. Also, this particular sample had been in − 20 °C storage for 2 years, which may have affected the DNA integrity. The occasional false-positive result in one *Plasmodium*-negative sample could have been the result of carry-over contamination from a *Plasmodium*-positive sample during the preparation of the dbPCR or NALFIA.

The LoDs of 100 p/μL for the pan-*Plasmodium* line and 10 p/μL for the *P. falciparum* line demonstrate the high sensitivity of the mini-dbPCR-NALFIA for low *falciparum* parasite densities. Although the LoD of extremely sensitive nested and qPCR techniques can go as low as 0.1 p/μL [34–37], most importantly, the mini-dbPCR-NALFIA is still significantly more sensitive for low *falciparum* parasitaemias than light microscopy and RDTs, which generally fail to detect infections below 50 to 200 p/µL [38]. As such, the assay will be able to diagnose the majority of symptomatic malaria patients in an endemic setting, who often present with a parasitaemia above 1000 p/μL [39–41]. On top of that, mini-dbPCR-NALFIA could potentially be used for screening and detection of asymptomatic *falciparum* cases with sub-microscopic infections [7, 42]. As no quantified non-*falciparum* samples were available for this study, additional evaluation of the LoD of the mini-dbPCR-NALFIA for these other *Plasmodium* species is warranted.

When analysing a *P. falciparum* blood dilution series and five malaria-negative blood samples, the mini-PCR-NALFIA showed a high accordance of 93.7%, demonstrating the robustness of the method. Discordant results were mainly observed for parasite densities < 10 p/μL, which are close to the LoD of the test. At such low *Plasmodium* DNA concentrations, stochastic variations tend to have a more prominent influence on the assay’s outcome. This phenomenon was also believed to be the main reason for the concordance being 84.6%. The laboratory experience of the different operators in the concordance evaluation ranged from basic to proficient. They were only given written and oral instructions, which was sufficient for them to correctly perform the mini-dbPCR-NALFIA. This observation underlined its simplicity and user-friendliness.

Compared to other molecular methods for malaria diagnosis, mini-dbPCR-NALFIA shares some characteristics with LAMP, which also has a simplified protocol with easy read-out and high accuracy for diagnosing malaria, including low density *falciparum* infections [43, 44]. However, LAMP currently has no multiplex capability and, therefore, cannot differentiate *Plasmodium* species in one reaction. This issue is not encountered with mini-dbPCR-NALFIA, a duplex assay that can distinguish *falciparum* malaria from infections with other *Plasmodium* species. To further evaluate the performance of the mini-dbPCR-NALFIA for diagnosis of (submicroscopic) infections with *P. vivax, P. malariae* and *P. ovale*., additional research is required, since this study tested only a limited number of non-*falciparum* malaria blood samples.

The adaptation of the assay described by Roth et al. [23] to operate on a portable, battery-powered mini16 thermal cycler has made it possible to run the dbPCR in harsh, resource-limited conditions of sub-Saharan Africa. Implementation in such settings is also supported by the stability of the dbPCR reagents, which did not show loss of performance after storage at 4 °C for 9 months [23]. Another strength of the mini-dbPCR-NALFIA is its affordability: the testing costs per sample are economical (0.30 USD for the dbPCR reagents, 2.80 USD per NALFIA test) and introduction of the mini16 greatly reduces the cost of the required equipment (800 USD per device). A planned economic evaluation will assess the cost-effectiveness of the mini-dbPCR-NALFIA in different endemic areas, compared to currently implemented malaria point-of-care diagnostics.

A limitation of the current mini-dbPCR-NALFIA is its inability to differentiate between the non-*falciparum* malaria species and identify mixed infections. Although the vast majority of malaria cases in Africa is caused by *P. falciparum*, the relative contribution of *P. vivax*, *P. malariae* and *P. ovale* infections in this region appears to be increasing [45–48]. Fortunately, the mini-dbPCR-NALFIA has a flexible design: an alternative format is currently under development, which will have a *P. falciparum* and a *P. vivax* test line. In the same way, the mini-dbPCR-NALFIA also has the potential to be modified to detect other blood-borne pathogens.

In areas with high malaria transmission, the mini-dbPCR-NALFIA could be a valuable alternative to RDTs, which are likely to suffer from false-positive results due to *P. falciparum* HRP2 antigen persistence in the blood after clearance of the parasites [13, 14, 49, 50]. Nevertheless, it is possible that a similar issue may arise for molecular diagnostic techniques: there have been a number of studies showing that PCR-based detection of *Plasmodium* DNA in blood can remain positive up to seven weeks after curative malaria treatment [51, 52]. This could either be caused by residual circulating DNA fragments or by a small subset of parasites with extended survival. Although this phenomenon could have its implications for the specificity of the mini-dbPCR-NALFIA, its relevance for the application of the assay as a field diagnostic remains a subject of further study.

## Conclusion

The mini-dbPCR-NALFIA is an easy-to-use method for sensitive and specific diagnosis of malaria. Compared to other simplified molecular diagnostics, it has the advantages that there is no need of prior sample processing and that differentiation of *P. falciparum* and non-*falciparum* infections is possible thanks to its duplex format. A handheld miniature thermal cycler makes the assay well-adapted to resource-poor conditions in malaria endemic regions. The high diagnostic accuracy and low LoD of the mini-dbPCR-NALFIA could make it a valuable tool in many malaria control programmes, especially for detection of asymptomatic and low-density cases in near-elimination areas. A phase-3 field trial is currently being conducted to evaluate the potential of the mini-dbPCR-NALFIA in different epidemiological settings.

## Data Availability

The datasets used and/or analysed during the current study are available from the corresponding author on reasonable request.

## Abbreviations

ACC: Accordance
CI: Confidence interval
CON: Concordance
dbPCR: Direct-on-blood PCR
Dig: Digoxigenin
DNA: Deoxyribonucleic acid
EDTA: Ethylenediaminetetraacetic acid
FAM: Fluorescein amidite
FITC: Fluorescein isothiocyanate
HRP2: Histidine-rich protein 2
LAMP: Loop-mediated isothermal amplification
LoD: Limit of detection
NAAT: Nucleic acid amplification technique
NALFIA: Nucleic acid lateral flow immunoassay
p/μL: Parasites per microlitre
PCR: Polymerase chain reaction
qPCR: Real-time quantitative PCR
RDT: Rapid diagnostic test
rRNA: Ribosomal ribonucleic acid
USD: United States dollars
